# Biobehavioral Implications of Covid-19 for Transplantation and Cellular Therapy Recipients

**DOI:** 10.3389/fimmu.2022.877558

**Published:** 2022-07-05

**Authors:** Jennifer M. Knight, Mallory R. Taylor, Kelly E. Rentscher, Elisabeth C. Henley, Hannah A. Uttley, Ashley M. Nelson, Lucie M. Turcotte, Natalie S. McAndrew, Hermioni L. Amonoo, Lathika Mohanraj, Debra Lynch Kelly, Erin S. Costanzo

**Affiliations:** ^1^ Department of Psychiatry and Behavioral Medicine, Medical College of Wisconsin, Milwaukee, WI, United States; ^2^ Department of Medicine, Medical College of Wisconsin, Milwaukee, WI, United States; ^3^ Department of Microbiology & Immunology, Medical College of Wisconsin, Milwaukee, WI, United States; ^4^ Department of Pediatrics, Division of Hematology/Oncology, University of Washington School of Medicine, Seattle, WA, United States; ^5^ Palliative Care and Resilience Program, Center for Clinical and Translational Research, Seattle Children’s Research Institute, Seattle, WA, United States; ^6^ Department of Psychiatry, Harvard Medical School/Massachusetts General Hospital, Boston, MA, United States; ^7^ Department of Pediatrics, University of Minnesota Medical School, Minneapolis, MN, United States; ^8^ College of Nursing, University of Wisconsin – Milwaukee, Milwaukee, WI, United States; ^9^ Froedtert Hospital, Froedtert & The Medical College of Wisconsin, Milwaukee, WI, United States; ^10^ Department of Psychosocial Oncology and Palliative Care, Dana-Farber Cancer Institute, Harvard Medical School, Boston, MA, United States; ^11^ Department of Psychiatry, Brigham and Women’s Hospital, Harvard Medical School, Boston, MA, United States; ^12^ Department of Adult Health and Nursing Systems, School of Nursing, Virginia Commonwealth University, Richmond, VA, United States; ^13^ Department of Nursing, University of Florida, Gainesville, FL, United States; ^14^ Cancer Population Science, University of Florida Health Cancer Center, University of Florida, Gainesville, FL, United States; ^15^ Department of Psychiatry, University of Wisconsin School of Medicine and Public Health, Madison, WI, United States

**Keywords:** transplantation and cellular therapy, biobehavioral, stress, Covid-19, outcomes

## Abstract

A growing body of literature has emphasized the importance of biobehavioral processes – defined as the interaction of behavior, psychology, socioenvironmental factors, and biological processes – for clinical outcomes among transplantation and cellular therapy (TCT) patients. TCT recipients are especially vulnerable to distress associated with pandemic conditions and represent a notably immunocompromised group at greater risk for SARS-CoV-2 infection with substantially worse outcomes. The summation of both the immunologic and psychologic vulnerability of TCT patients renders them particularly susceptible to adverse biobehavioral sequelae associated with the Covid-19 pandemic. Stress and adverse psychosocial factors alter neural and endocrine pathways through sympathetic nervous system and hypothalamic-pituitary-adrenal axis signaling that ultimately affect gene regulation in immune cells. Reciprocally, global inflammation and immune dysregulation related to TCT contribute to dysregulation of neuroendocrine and central nervous system function, resulting in the symptom profile of depression, fatigue, sleep disturbance, and cognitive dysfunction. In this article, we draw upon literature on immunology, psychology, neuroscience, hematology and oncology, Covid-19 pathophysiology, and TCT processes to discuss how they may intersect to influence TCT outcomes, with the goal of providing an overview of the significance of biobehavioral factors in understanding the relationship between Covid-19 and TCT, now and for the future. We discuss the roles of depression, anxiety, fatigue, sleep, social isolation and loneliness, and neurocognitive impairment, as well as specific implications for sub-populations of interest, including pediatrics, caregivers, and TCT donors. Finally, we address protective psychological processes that may optimize biobehavioral outcomes affected by Covid-19.

## Intersecting Adversities: Transplantation and Cellular Therapy and the Covid-19 Pandemic

The Covid-19 pandemic, caused by the severe acute respiratory syndrome coronavirus 2 (SARS-CoV-2), has claimed the lives of over 5 ½ million people worldwide as of January 28^th^, 2022 (1, 2). Resulting public health measures have significantly altered the social environment, increasing social isolation and feelings of loneliness (2). The pandemic has also precipitated economic hardship and financial strife (3). These social stressors have adversely affected mental health, particularly within socially and physically vulnerable populations (4).

Transplantation and cellular therapy (TCT) recipients comprise an immunocompromised group at greater risk for Covid-19 infection and poorer outcomes, including increased risk for mortality (5). They are already vulnerable due to their intense treatment regimens accompanied by prolonged hospitalizations, high risk of medical complications, and immediate and long-term medical comorbidities (6, 7). TCT recipients also have heightened risk for clinically significant distress and poor psychological function due to pandemic conditions (2, 8, 9).

A growing body of literature documents the importance of biobehavioral processes – defined as the interaction of behavior, psychology, socioenvironmental factors, and biological processes – for clinical outcomes among TCT patients (10, 11). The summation of both the immunologic and psychological vulnerability of TCT patients renders them particularly susceptible to adverse biobehavioral sequelae associated with the conditions of the current global pandemic, potentially affecting clinical and patient-reported outcomes.

### Biobehavioral Processes in TCT

Cancer development and progression, as well as TCT treatment, alter innate and adaptive immune function (12). The significant stress associated with a cancer diagnosis, treatment, and accompanying life disruption can also adversely affect immune function (13, 14). Specifically, stress and adverse psychosocial factors alter neural and endocrine pathways that ultimately affect gene regulation in immune cells (15–19). This signaling is primarily mediated through the sympathetic nervous system (SNS) (20, 21) and hypothalamic-pituitary-adrenal (HPA) axis (22). These biobehavioral signals affect the tumor microenvironment through mechanisms including the promotion of tumor-cell growth and spread, angiogenesis, and alterations in antibody and cytokine production profiles and cell trafficking (22). Inflammation due to tissue damage from intensive conditioning therapy and the occurrence of infections contributes to dysregulation of neuroendocrine and CNS function, resulting in the symptom profile of depression, fatigue, sleep disturbance, and cognitive dysfunction (12, 22). In addition, inflammation plays a central role in the pathogenesis of graft-versus-host disease, a debilitating syndrome that attacks organs following allogeneic HCT resulting in a cytokine storm of inflammatory activity, as well as the CRS common following CAR T cell therapy, further exacerbating neuropsychiatric symptoms (23, 24). Gene expression profiling studies of people exposed to chronic threat have identified a “conserved transcriptional response to adversity” (CTRA) in circulating immune cells (25). CTRA expression is mediated by SNS activity and associated with adverse TCT outcomes (26) and may represent a pathway by which pandemic-associated stress influences clinical TCT outcomes.

### Psychosocial Effects of Covid-19 for TCT Patients

TCT patients are already at an increased risk for adverse psychological effects, including depression, anxiety, and persistent distress (13, 27). The Covid-19 pandemic has exacerbated stress, with cancer patients reporting increased depression, anxiety, and distress related to their physical health since the onset of the pandemic (8, 9, 28–32). For TCT patients, care disruptions and delays can be particularly stressful (10, 33). Because of the increased risk of severe Covid-19, many TCT patients also have heightened fear of contracting the SARS-CoV-2 virus (34, 35). The pandemic-induced limitations on travel creates additional distress for patients who must travel long distances to receive care at the few TCT centers in the U.S. and around the world (36). Social isolation and quarantine requirements during post-TCT recovery already cause distress for TCT recipients (37), which is worsened by more extensive isolation related to Covid-19 precautions (7). The widespread economic hardship associated with the Covid-19 pandemic can also exacerbate the adverse financial impact of TCT (38–40).

### Biological Effects of Covid-19 Disease Processes Relevant to TCT

SARS-CoV-2 targets the angiotensin-converting enzyme 2 expressed by airway epithelial cells, alveolar epithelial cells, vascular endothelial cells, and macrophages in the lung (41–44). The virus then penetrates the host cell membrane wherein viral RNA is released and gains access to the cellular machinery necessary for self-replication and release, spreading infection and damaging host cells (41, 42). The body’s initial innate immune response is characterized by the inflammatory actions of alveolar macrophages and recruitment of T lymphocytes, monocytes, and neutrophils. In a healthy immune response, this inflammation is carefully regulated and works in tandem with the body’s slower adaptive immune mechanisms—characterized by the action of CD4^+^ and CD8^+^ T cells and the release of antibodies by B cells—to eliminate infected cells and neutralize the virus. However, most patients with severe Covid-19 infection instead experience a dysfunctional immune response, in which unchecked inflammation continues and becomes widespread, driving systemic cytokine storm and eventually resulting in pulmonary edema, pneumonia, possible microthrombus formation, and multi-organ damage (42–44). TCT patients are especially vulnerable to severe infection and associated complications.

Further, SARS-CoV-2 infection impacts the central nervous system (CNS) directly through several mechanisms. Viral penetration triggers a neuroinflammatory reaction that leads to microglial activation, triggering a demyelinating process that acts as a primary etiology for encephalopathy (45). In preclinical models, coronavirus particles are capable of invading cells of the cortex, hypothalamus, thalamus, amygdala, basal ganglia, pyriform cortex, and brain stem while proliferating in the limbic structures, which can ultimately lead to behavioral changes (46).

### Summary

**Figure 1:**
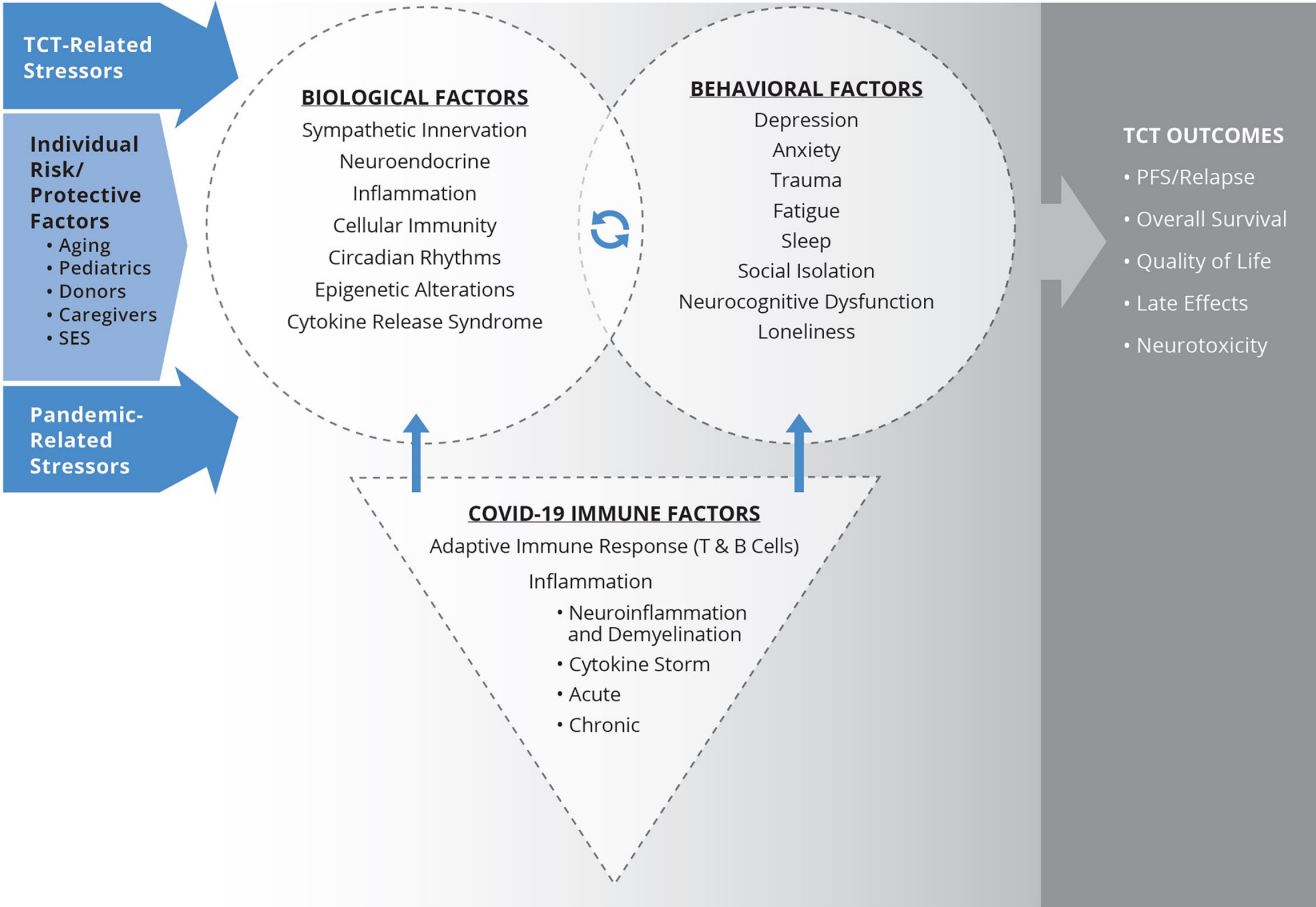
Biobehavioral model illustrating Covid-19 impact for TCT patients. Biological processes affected by TCT, including inflammation and disrupted circadian rhythms, alter central nervous system pathways that evoke behavioral symptoms. Stress-related behavioral factors activate HPA and SNS axes and are associated with downstream immunologic and genomic alterations. The products of these pathways, as well as direct sympathetic innervation of the bone marrow microenvironment, can modulate immune recovery and inflammation, potentially influencing clinical outcomes following TCT. Behavioral factors also directly relate to quality of life and late effects. Stress and adversity associated with the Covid-19 pandemic can exacerbate biological and behavioral impacts of TCT and biobehavioral mechanisms associated with poorer clinical outcomes. Similarly, infection with SARS-CoV-2 can also adversely affect biobehavioral factors and relationships. The paper further considers the intersection of Covid-19 impacts and individual differences in risk and protective factors, including age, SES, caregiver status, and donor characteristics relevant to biobehavioral processes and TCT outcomes.

In this article, we expand upon the above overviews to examine intersections of inflammation, immunology, aging, psychology, infection, neuroscience, cancer, viruses, stress, Covid-19 pathophysiology, and TCT processes that can influence TCT outcomes. The major relationships and pathways described are summarized in **Figure 1**. The goal is to provide an overview of the significance of biobehavioral factors in understanding the relationship between Covid-19 and TCT. Specific symptoms and states that will be discussed include depression, anxiety, fatigue, sleep, social isolation and loneliness, and neurocognitive impairment (NI). We will also discuss specific implications for sub-populations of interest within the TCT context, including pediatrics, caregivers, and donors. Finally, we address protective psychological processes that may optimize biobehavioral outcomes affected by Covid-19.

## Stress-Related Biobehavioral Factors

### Depression and Anxiety

Patients receiving TCT are at risk for short and long-term neuropsychiatric toxicities, including anxiety, depression, and trauma-related stress symptoms. These symptoms may be a consequence of underlying disease, the treatment itself, and the complex psychosocial and environmental factors that are interwoven into the cellular therapy experience and may also be exacerbated by the Covid-19 pandemic. The prevalence of psychological symptoms in hematopoietic cell transplant (HCT) patients is high compared to the general population, typically with a peak in the peri-transplant period and improvement over time (47). In a study of 90 HCT patients, nearly 30% met criteria for PTSD and 43% reported clinically significant depression symptoms at 6 months post-transplant (48). In survivors of pediatric HCT, the prevalence of clinically significant anxiety is estimated to be between 16-37%, which is higher than age-matched controls and siblings (49, 50). Less is known about prevalence of these symptoms in patients receiving chimeric antigen receptor (CAR) T cell therapy, but early data suggest nearly 50% of patients experience at least one clinically meaningful negative neuropsychiatric outcome (51).

#### Psychosocial Effects of Covid-19

The impact of pandemic-related personal and societal disruptions on cellular therapy patients’ mental health is still being investigated. Covid-19 survivors have consistently reported anxiety, depression, and trauma-related symptoms following their diagnosis (52). A recent study compared patient-reported depression, anxiety, and PTSD symptoms between HCT recipients who received treatment just prior to (March 2019-January 2020) and during (March 2020-January 2021) the Covid-19 pandemic (7). This study did not find any differences in psychological symptoms between the two cohorts. Covid-19-era patients described negative impacts of the pandemic on their HCT recovery, including increased isolation during their hospitalization and heightened distress about being infected by SARS-CoV-2. They also articulated positive downstream effects of the pandemic, including increased access to their support network through adaptation of social activities to virtual platforms and comfort in mandated public health safety measures (7).

#### Biological Effects of Covid-19

Symptoms of depression, anxiety, and traumatic stress all have well-documented neuroimmune correlates relevant to cellular therapy patients (12, 33, 53). SNS-mediated beta-adrenergic signaling (activated during ‘fight-or-flight’ states such as anxiety or the hypervigilance of PTSD) influences stem cell migration, homing, and proliferation (54, 55). In CAR T cell therapy, cytokine release syndrome (CRS) and related neurotoxicity syndromes lead to profound peripheral and sometimes central inflammation, with demonstrated downstream neuropsychiatric sequelae (51, 56). There is overlap in the pathophysiology of neuroimmune-mediated symptomatology between HCT and CAR T cell therapy and Covid-19 disease. Thus, it is important to consider the interrelated psychoneuroimmune effects of a SARS-CoV-2 infection in patients with baseline aberrant immune repertoires. The SARS-CoV-2 virus can elicit an extreme, life-threatening acute inflammatory response, but even mild infections can result in significant neuroinflammation and myelin loss (57). The resulting symptoms, which can persist beyond the acute infection period as a post-Covid condition (including “Long Covid”) share some features with neurotoxicity syndromes or chemotherapy-associated neuropsychiatric dysfunction (57). Close attention to new or worsening neuropsychiatric symptoms in cellular therapy patients following a Covid-19 diagnosis is warranted.

#### Summary

Pandemic-related individual and societal disruptions have had almost universally negative effects on global mental health, but we are still learning about specific effects on mood symptoms in patients receiving HCT or CAR T cell therapy. There also may be some systemic adaptations to the pandemic that have the potential to positively impact patients’ experience and mood.

### Fatigue and Sleep

Fatigue and sleep disruption are some of the most prevalent and distressing symptoms experienced by TCT recipients before, during, and after treatment (58–60). Fatigue tends to be ubiquitous during hospitalization for HCT but may gradually remit for patients during the 100-day post-transplant recovery period. Although fatigue often improves over time, evidence suggests that as many as 35-64% of HCT recipients go on to experience persistent fatigue during long-term post-transplant recovery (61–63). A similar pattern exists for sleep problems, with over 50% of patients experiencing sleep disruption prior to transplant and up to 43% following discharge home (27, 59, 64). In addition, early work characterizing patient-reported outcomes among patients receiving CAR T cell therapy suggests that fatigue and sleep disruption do not improve even by six months following treatment (60).

Persistent fatigue and sleep disruption not only disrupt engagement in everyday routines, but have been linked to self-reported cognitive problems (51, 65, 66), diminished social adjustment and engagement (51, 67, 68), unemployment status (69, 70), and impaired mood in TCT patients (27, 71). There is also evidence to suggest that fatigue and sleep disruption may predict clinical outcomes following HCT, with findings from a recent study revealing that high pre-HCT sleep disruption predicted an increased risk of disease recurrence and poorer survival, and that greater post-HCT fatigue interference was also associated with poorer survival (72).

The contributors to fatigue and sleep disruption among TCT recipients are multifactorial, with demographic, biological, medical, psychosocial, and behavioral influences (10, 11, 53, 73, 74). While evidence is preliminary, the Covid-19 pandemic is likely to influence the symptoms experienced by TCT patients across these multifactorial domains.

#### Psychosocial Effects of Covid-19

The protracted recovery period following TCT, in which patients are immunosuppressed and endure protective isolation, imposes drastic and prolonged lifestyle adaptations (e.g., social isolation, activity reduction) that may exacerbate fatigue and sleep disruption long-term. These adaptations are amplified in the context of the Covid-19 pandemic. In addition, Covid-19 related stressors are associated with a heightened and prolonged state of psychological stress that can initiate or contribute to symptoms of fatigue and sleep disruption (31, 75). However, a recent report revealed no differences in pre-HCT fatigue before or during the Covid-19 pandemic (7). Findings highlight the importance of capturing the symptom experience across the TCT trajectory as well as the value in understanding sources of both vulnerability and resiliency from the pandemic and effects on biobehavioral symptoms.

#### Biological Effects of Covid-19

Both psychological stress and viral infection can disrupt the HPA system and affect fatigue and sleep disruption directly through alternations in cortisol or indirectly *via* modulation of immune pathways (2, 10, 76). Further, HPA axis dysregulation and inflammatory processes can disrupt circadian rhythms, which has been linked to poor QOL among HCT recipients (77) and heightened fatigue and sleep disruption among other cancer populations (78). As noted previously, inflammation is common in the TCT setting (23) and occurs in response to infection with the novel SARS-CoV-2 infection (5). This inflammatory cascade can affect CNS circuitry leading to behavioral responses associated with the withdrawal and conservation of energy, including fatigue and sleep disturbance (79–81). In HCT patients, fatigue and sleep disruption are both associated with higher circulating levels of interleukin (IL)-6, particularly in earlier post-transplant recovery (62, 82–85). There is evidence of a similar hyperinflammatory state occurring amongst a subgroup of patients with severe SARS-CoV-2 infection that may share underlying pathophysiology and subsequent biobehavioral symptom profiles (86, 87). It remains unclear whether this mechanism influences the prolonged biobehavioral symptoms exhibited in “post-Covid-19 syndrome” (88).

#### Summary

A common framework used to understand fatigue and insomnia is a biopsychosocial model of predisposing, precipitating, and perpetuating factors (89–91). Predisposing factors refer to characteristics that increase an individual’s vulnerability to symptom development. Precipitating factors refer to triggers leading to the onset of fatigue or insomnia episodes. Finally, perpetuating factors are features that contribute to the maintenance or exacerbation of symptoms over time. With the added complications of the Covid-19 pandemic, consistent use of this framework to guide research efforts may prove useful for advancing the biobehavioral science of fatigue and sleep disruption in the TCT context.

### Social Isolation and Loneliness

Social isolation and loneliness have been increasingly recognized as a public health priority due to their effects on mental and physical health (92) and have been a significant concern during the Covid-19 pandemic. Although social isolation and loneliness are sometimes correlated, social scientists make conceptual and empirical distinctions between the two constructs (93). *Social isolation* is a lack of (or infrequent) social contact that may occur when an individual lives alone or has few social ties, whereas *loneliness* is a subjective feeling of social disconnection that results from a discrepancy between an individual’s actual and desired social relationships (94). To date, more than a dozen studies have investigated loneliness in cancer patients and survivors, including TCT recipients, whereas social isolation has received little empirical attention in these populations. A meta-analysis conducted prior to the pandemic suggested that 32-47% of cancer survivors experience loneliness (95). In a study of HCT recipients, 39% of the sample and 70% of the subset of patients with elevated psychological distress reported feeling lonely (96).

#### Psychosocial Effects of Covid-19

Due to immunocompromised status and increased risk for severe complications from Covid-19 (97–99), cancer patients have been particularly advised to physically isolate from others during the pandemic. The degree of physical isolation required during the pandemic is in clear contrast to the typical recommendation for cancer patients to engage their social and support networks, including family members and other social ties, community organizations, and places of worship (100, 101). For TCT recipients, the routine physical isolation that occurs during hospitalization and the early recovery period may persist longer and be even more restrictive during Covid-19 due to no-visitor hospital policies, contributing to social isolation and loneliness (102). Indeed, several studies conducted during the pandemic estimate the prevalence of loneliness in cancer patients and survivors is 48-53% (103–107), an increase from pre-pandemic estimates. In addition, a handful of longitudinal studies found that loneliness in cancer patients increased during the pandemic and was associated with poorer mental health (28, 108–110). This research identified active treatment, younger age, non-white, unmarried/unpartnered, living alone, and having a pre-existing mental health condition as risk factors for loneliness during the pandemic (104, 106, 107). Although one qualitative study suggested that HCT recipients experienced decreased social connection and support during the pandemic (36), empirical data on loneliness among TCT patients specifically are not yet available. However, one study reported that blood cancer survivors were more likely to report being lonely and had a larger increase in depression symptoms during the pandemic as compared to other cancer populations (28).

A meta-analysis also found that social isolation and loneliness were associated with a 29% and 26% increased risk of all-cause mortality, respectively, which is comparable in magnitude to well-established risk factors such as obesity and smoking (111). Among cancer patients and survivors, loneliness and has also been associated with higher pain, fatigue, anxiety, and depression, as well as poorer cognitive function and health-related QOL (112–116). In two studies with breast cancer survivors, social isolation (i.e., few intimate contacts and group memberships) was associated with increased risk for cancer recurrence, breast cancer mortality, and all-cause mortality, and survivors who were isolated were more likely to smoke and engage in less physical activity than those who were socially integrated (117, 118). To date, however, the potential impact of social isolation and loneliness, including during the Covid-19 pandemic, on transplant-related and other health outcomes among TCT recipients has not been investigated, representing an important research direction.

#### Biological Effects of Covid-19

Social isolation and loneliness are forms of chronic stress that also initiate a stress-signaling cascade characterized by increased SNS and HPA axis activation that can affect the expression of immune response genes, including those involved in inflammation and the anti-viral response (119). In non-cancer populations, loneliness has been associated with peripheral and transcriptomic indicators of SNS and HPA axis activity, inflammation, and antiviral immunity, including elevated urinary norepinephrine levels, altered diurnal cortisol patterns (e.g., cortisol awakening response), increased peripheral markers of inflammation (e.g., IL-6), and elevated inflammatory and reduced glucocorticoid receptor and antiviral gene expression (for review, see Cacioppo and Cacioppo, 2018) (120). Inflammation reciprocally feeds back to the brain to further influence feelings of loneliness (121, 122). Other research has found that social isolation and loneliness are associated with lower influenza antibody levels following vaccination (123) and greater symptoms of infection following exposure to the common cold virus (124, 125), with potentially important implications for Covid-19 vaccination response and risk for infection among immunocompromised TCT recipients.

In a study with breast cancer survivors, lonely patients showed greater synthesis of IL-6 and IL-1b in peripheral blood mononuclear cells stimulated with lipopolysaccharide following an acute stressor in the laboratory compared to those who were less lonely (115). Another study with colorectal cancer patients found that those who were lonely had greater expression of vascular endothelial growth factor in tumor tissues following surgery; however, self-reported loneliness did not show an association (126). Finally, research with breast and ovarian cancer survivors suggests that women reporting lower levels of emotional closeness in their relationships, which is conceptually similar to loneliness, had pro-metastatic molecular profiles (e.g., gene expression patterns consistent with epithelial-mesenchymal transition) in primary tumors and exosomes (127–129).

#### Summary

Together, these findings suggest that social isolation and loneliness may affect key stress response and immune pathways, including those related to cancer progression. Given that social isolation and loneliness may be amplified as a result of the Covid-19 pandemic, it will be important for researchers and clinicians working with TCT populations to consider these potential risk factors that may interact with TCT treatments to influence transplant-related and other behavioral and patient-reported outcomes (130).

### Neurocognitive Impairment

NI, also referred to as neurocognitive dysfunction or cognitive dysfunction, is a complex issue with varying manifestations affecting multiple domains of cognition, including memory, attention, concentration, executive function, processing speed, and learning (131). Risk factors for NI include underlying disease burden, radiation and chemotherapy, and immunologic therapies (132, 133). NI is a top concern for TCT patients and caregivers, with 40- 60% of adult HCT survivors reporting NI five or more years post HCT (74, 134–136) (137, 138). Early data from CAR T cell therapy recipients demonstrate that approximately 37.5% reported NI at 1 to 5 years post-therapy, including problems with memory, word finding, and concentration (51). NI has also been observed in 15-80% of sampled Covid-19 participants across several studies (139). Risk factors for NI in Covid-19 patients are not yet known, although there is some evidence that those with more severe or complicated infections are at greater risk (140).

#### Psychosocial Effects of Covid-19

Psychological stress is associated with impaired cognitive function, presenting a point of vulnerability for biobehavioral complications of Covid-19 in TCT recipients. Among cancer patients, 91.5% of those classified as experiencing extremely high stress had NI, as compared to an incidence of 75% among cancer patients more broadly (31). Stressors associated with the Covid-19 pandemic may also exacerbate NI in the TCT population, although this is yet to be formally investigated. Early data among CAR T cell recipients shows that blood kynurenine concentrations – a molecular marker linking inflammation and the brain – are increased for those reporting more depression (141). In turn, increased kynurenine and its metabolites were further predictive of greater neurotoxicity (141), suggesting one plausible biologic mechanism linking CNS function to NI that could be exacerbated by pandemic conditions.

#### Biological Effects of Covid-19

Inflammation, HPA axis dysfunction, and altered monoamine neurotransmission may contribute to NI following treatment (142–155). Among HCT patients, increases in circulating markers of inflammation, including IL-6 and soluble tumor necrosis factor (TNF) receptor II, are associated with worsening cognitive function from pre-HCT to 90 days following HCT, while decreases in C-reactive protein are associated with better cognitive performance (133).

The two most common toxicities associated with CAR T cell therapy are CRS and immune effector cell-associated neurotoxic syndrome (ICANS), both of which are unique to CAR T vs. HCT and are associated with neurocognitive sequela (51). Inflammatory cytokines, including interferon gamma, IL-6, IL-8, IL-10, and monocyte chemoattractant protein-1, are elevated in individuals progressing to severe CRS (143). ICANS typically - though not always - presents in conjunction with CRS, suggesting overlapping but not identical pathology (144).

Given that inflammation is a significant component of Covid-19 pathophysiology and directly contributes to NI, it is plausible that infection with SARS-CoV-2 may additively or synergistically contribute to NI among TCT recipients, although there is not yet research addressing this. In particular, the cytokine storm associated with Covid-19 may contribute to worse NI among TCT recipients. This effect may be compounded for CAR T cell recipients who concurrently experience CRS or ICANS, though this remains to be investigated. Given that TCT patients become more severely ill if infected with SARS-CoV-2, they may also be more likely to experience adverse neuropsychiatric sequelae and NI based on what is known about NI outcomes with severe or complicated Covid-19.

#### Summary

Data on the pathophysiology of Covid-19, TCT therapies, the neuropsychiatric sequelae of SARS-CoV-2 infection and pandemic restrictions, and the overlapping biologic etiologies therein strongly support the need for further investigation of the impact of Covid-19 on NI in the TCT population.

## Subpopulations of Interest Within the Context of TCT

### Pediatrics

Cellular therapies, which both create and harness extreme immune dysregulation, have revolutionized the care of many types of pediatric cancer (53, 145, 146). Young patients receiving TCT in the era of Covid-19 are positioned to experience the convergence of profound social, neuropsychiatric, and immune disturbances in a distinct way. The Covid-19 pandemic has undeniably affected children and adolescents differently than older adults. This applies to both the pathophysiology of SARS-CoV-2 infection in younger persons, as well as the psychosocial implications of the large-scale societal disruptions. Not only do children and adolescents undergoing TCT face the same Covid-19-related risks as other children, but they must also cope with the immense burdens of cancer and treatment.

Healthy children and adolescents typically experience less severe disease after infection with SARS-CoV-2 compared to adults (147). However, children and adolescents with cancer may be at increased risk of poor outcomes. In a global cohort of pediatric patients with cancer or HCT, severe or critical illness occurred in nearly 20% of patients, and 4% died from Covid-19-related complications (148). Indirect complications of Covid-19 in pediatric TCT patients mirror that of adults, including delayed diagnoses, treatment interruptions, loss of caregivers, and postponement or even withdrawal of cellular therapies due to strained healthcare resources (149, 150). Most pediatric cellular therapy patients are treated as part of a clinical trial, and thus pandemic-related disruptions in research infrastructure can significantly impact the clinical care of these patients (151).

At the pathophysiologic level, there are commonalities between some of the unique manifestations of Covid-19 in children and the sequelae of cellular therapy. For example, multisystem inflammatory syndrome in children is a rare but serious entity characterized by hyperinflammation and end-organ dysfunction (152). Dangerous over-activation of the immune system is also a hallmark of CRS and ICANS following CAR T cells (56, 153). Given the known associations between severe inflammation and adverse neuropsychiatric outcomes (56, 79), close evaluation of these patients is warranted. For children and adolescents, there are likely significant implications of experiencing these inflammatory insults during critical developmental periods. Indeed, there are reports of prolonged neurocognitive deficits and adverse QOL in pediatric Covid-19 survivors (154, 155). There is a scarcity of data regarding severe Covid-19 in pediatric patients with baseline dysregulated immune systems, and the long-term neuropsychiatric sequelae of severe inflammation associated with SARS-CoV-2 infection in young cellular therapy patients should be an area of active research focus.

Not only can the inflammatory response drive poor neuropsychiatric outcomes, but behavioral and socioenvironmental factors can also influence the immune system (11, 53, 156). Extreme isolation, interruption of school and peer supports, health risks, exacerbation of economic hardships, compounded family stress, and death of friends and family have culminated in a youth mental health crisis during the pandemic (157, 158). Young patients with cancer are experiencing alarmingly high rates of anxiety and depression, with 60-80% of patients reporting worsening of symptoms since the onset of the pandemic (105, 159). Cellular therapy patients may be at even higher risk of poor mental health outcomes. These types of physical and existential threats have been associated with downstream immune effector cell function, which have clear implications for cellular therapy patients (160–162). Thus, the overlay of pandemic-related distress onto cancer-related psychological and emotional challenges at critical developmental periods for young patients may have implications at the biologic level.

#### Summary

The Covid-19 pandemic has introduced a plethora of new stressors that are unique to young cellular therapy patients. Important questions remain regarding pediatric-specific immune responses to SARS-CoV-2 infection, especially for patients who may have abnormal immune repertoires. Applying a biobehavioral lens to research and clinical care as the pandemic continues to unfold may offer valuable insights and tools in the care of this particularly vulnerable TCT subgroup during this unprecedented time and as we prepare for the future (53, 163).

### Caregivers

Family caregivers play a critical role in the well-being of patients undergoing TCT. Caregiver involvement has been associated with lower patient distress and even improved patient survival (164–166). Caregiver distress is also associated with poorer patient response to treatment (167), and caregiver well-being is associated with better patient QOL (168). Therefore, the biological and psychological effects of pandemic-related stress for caregivers has the potential to translate to downstream adverse TCT patient outcomes (167, 169–173). The Covid-19 pandemic undoubtedly imposes additional stress on TCT caregivers; however, little research has directly explored this (174–176). Meanwhile, the current body of TCT caregiver biobehavioral research (170, 177–181) – albeit limited – helps offer insight into how the unique stressors of a global pandemic likely have consequences not only for caregivers, but potentially implications for patient outcomes as well.

#### Psychosocial Effects of Covid-19

Prolonged stress contributes to adverse psychological outcomes for TCT caregivers, including anxiety and depression, family strain, and spiritual and existential distress (172, 173, 182, 183) (184–186). TCT caregivers show even higher levels of stress than do dementia caregivers, with 30% of HCT caregivers meeting criteria for clinically significant psychological distress (187–189).

The Covid-19 pandemic adds to the caregiving burden and increases distress for TCT caregivers. Caregivers have less access to support during the pandemic, thereby increasing their caregiving responsibilities and burden (188). Since the pandemic started, caregivers report more anxiety, depression, fatigue, and sleep disturbance in addition to lower social participation and poorer financial well-being than non-caregivers (190). Adults caring for a parent with hematologic cancer reported distress related to reduced in-person health care, increased uncertainty, and social isolation during pandemic conditions (175). Parents supporting their children through HCT during Covid-19 shared that the HCT experience uniquely prepared them to cope with the pandemic; however, it also diminished their access to coping resources, reduced their social support, and amplified their germ-related fears (174). Further, parents of children with hematologic cancers reported feeling more vulnerable during the pandemic (176). Notably, increased isolation to protect their loved one from Covid-19 may contribute to TCT caregiver loneliness (191).

#### Biological Effects of Covid-19

While the biological effects of Covid-19 on caregivers have not been specifically studied, stress-related dysregulation of the HPA axis has been observed among TCT caregivers and may be exacerbated by Covid-19 related stressors (170, 180, 181, 189, 192, 193). Caregiver stress is also associated with enhanced expression of the proinflammatory CTRA gene profile (26, 138, 177). In a study of caregivers of patients with colorectal cancer, social isolation and lack of social support were associated with increased CTRA gene expression, which has implications for social isolation experienced during the Covid-19 pandemic (177).

#### Summary

The prolonged stress of Covid-19 likely contributes directly to adverse psychological and biological sequelae of TCT caregivers, as well as indirectly to TCT recipient outcomes, through the manifestation of caregiver stress and social isolation. Further research is needed to evaluate the intersection of burdens of TCT caregiving and pandemic conditions with the goal of optimizing caregiver and patient outcomes.

### Donors

The impact of Covid-19 on the clinical and physiologic status of hematopoietic cell donors and the downstream effects on HCT recipients has not been well-described to date. However, there are several plausible impacts that warrant further evaluation. First, socioeconomic hardships associated with Covid-19 may affect the ability of individuals to volunteer as donors because of financial, work, or childcare constraints, although this has not yet been confirmed. Even when willing to donate, finding times for confirmatory testing and donation pose greater challenges and barriers than prior to the Covid-19 pandemic.

Beyond the logistical donor issues, the downstream effects of pandemic-related stress alter the bone marrow microenvironment where hematopoietic stem cells are produced, affecting the donor stem cell product with short- and long-term consequences for the HCT recipient. HCT recipients who received fully-matched peripheral blood stem cells from donors with greater socioeconomic disadvantage were observed to experience inferior overall survival (OS), disease-free survival (DFS), and transplant-related mortality (TRM) (137). These findings were independent of donor or recipient race and of recipient SES. Findings highlight the adverse effects of socioeconomic stress down to the level of the hematopoietic cell, and even after the cells engraft in a new host. The biologic underpinnings of these associations are attributed to a pro-inflammatory state induced by chronic stress (194, 195). However, another study found that donor inflammatory cytokine and adipokine levels (IL-6, IL-1b, TNF-a, leptin, adiponectin, and ST2) were not associated with recipient outcomes (196). Novel work is investigating associations between donor CTRA gene profile and recipient outcomes (197); however, results of those analyses are not yet available (137).

As the Covid-19 pandemic persists, further attention to its impact on HCT donors is needed. The ramifications of donor availability or alterations in hematopoietic cell function and health secondary to pandemic stressors may have broad-reaching implications for HCT outcomes.

## Biobehavioral Factors That Confer Resilience

While the focus thus far has been on processes of stress and impairment, it is also important to identify biobehavioral processes that mitigate stress and optimize adjustment and recovery. Responses to adversity can be characterized by *survival with impairment*, a pattern of persistently compromised functioning, which is distinguished from *resilience*, defined as a return to normal or baseline functioning, which is then further distinguished from *thriving*, described as exceeding one’s original level of functioning (198, 199). Identifying factors that confer psychological and biological recovery and resilience to TCT and Covid-19-related impacts has translational relevance for the development of effective interventions.

### Psychosocial Factors That Confer Resilience

At the time of writing, we are not aware of published data regarding resilience factors in the context of intersecting stressors associated with TCT and the Covid-19 pandemic. The TCT literature, however, highlights the salubrious influence of supportive social relationships, which are associated with more optimal psychological and physical function (200, 201). A recent study of cancer survivors suggested that older age may mitigate the impact of Covid-19 related stressors, with older adults redeploying and repurposing coping previously used for cancer and other health concerns (202). A recent study found that successfully navigating a cancer diagnosis and treatment may have prepared cancer survivors for the existential distress of the pandemic and conferred additional resilience (203).

How TCT patients approach both external stressors and their internal responses to stress can also affect outcomes. HCT patients who respond to difficult thoughts and emotions mindfully, without judgement or reactivity, and those patients who actively cultivate a sense of meaning and purpose, show better psychological adjustment and physical function (204, 205). Coping approaches that involve active engagement with stressors (e.g., planning, problem-solving) and internal experience (e.g., emotional processing) also facilitate better psychological function and fewer physical symptoms among HCT patients (200, 201). Consistent with these findings, a large study of adults in the US and Canada found that active, approach-oriented coping strategies in response to Covid-19 pandemic stressors were associated with more optimal mood and QOL, whereas coping strategies characterized by avoidance (of the stressor itself and one’s emotions and thoughts) were associated with greater mood disturbance and poorer QOL (154). Cognitive behavioral and mindfulness-based interventions that help TCT patients to engage with stressors and emotion may be particularly fruitful in facilitating recovery and well-being for those facing the dual challenges of TCT and the Covid-19 pandemic.

### Biological Factors That Confer Resilience

Several key mechanisms of biological resilience to stress have been well documented and may be of particular relevance for patients facing intersecting stressors associated with TCT and Covid-19. First, the HPA axis plays an important role in resilience to stress (206). Postsynaptic receptors of the hippocampus, such as G-protein coupled GABA_B_ receptors, are important in stress regulation and confer resilience to stress-induced anhedonia and social avoidance. The vagus nerve is also important in autonomic control of cardiac activity and plays a vital role in mind-body interactions that involve the immune and endocrine systems as well as neurocognitive effects. In addition, the ability to maintain heart rate variability (HRV) during stressful events is associated with a faster and more robust recovery of immune function and optimizes endocrine and cardiovascular responses to stress (207) (208)., Finally, gene expression variations and chromatin alterations in the ventral tegmental area (midbrain) and nucleus accumbens (forebrain) have been observed in resilient animals (209, 210); identification of such ‘resilience genes’ may facilitate targets for pharmacologic and behavioral interventions.

Biomarker discovery and risk stratification have also yielded insights into biological factors that confer resilience to stress and psychiatric disorders. Studies have only shown weak to moderate associations of genes related to the HPA axis or serotonergic systems with resilient phenotypes. Neuropeptide Y regulates HPA axis activity and has been shown to reduce anxiety following intranasal delivery (211, 212). Epigenetic alterations (DNA methylation, histone modifications, non-coding RNAs) may also influence recovery and resilience (213), with differential DNA methylation patterns observed in resilient individuals versus those who developed PTSD following a traumatic event (214). These patterns were related to the HPA axis, inflammatory pathways, and methylation function (214–216).

Taken together, this growing body of work suggests novel therapeutic targets that have potential to mitigate stress associated with the Covid-19 pandemic and optimize the biological and psychological recovery from TCT. With respect to pharmacologic approaches, propranolol, a non-selective beta antagonist, has the potential to enhance biological resilience under conditions of stress and adversity; a recent study found that propranolol reduces CTRA gene expression and inhibits cellular and molecular pathways associated with adverse outcomes in HCT recipients (217). Behavioral interventions including physical activity, slow breathing, and meditation also improve vagal function and facilitate recovery from stress through immunologic, endocrine, and psychologic mechanisms (218). These efficient and cost-effective interventions can be delivered in the context of the demands and limitations associated with the pandemic and TCT and may confer long-lasting resilience throughout the recovery period, though the specific impacts have yet to be directly investigated.

## Summary and Future Directions

The Covid-19 pandemic has dramatically changed not only the current world conditions, but the clinical practice of TCT. The intersection of behavioral, psychological, socioenvironmental, and biological processes associated with both Covid-19 and TCT will likely have far-reaching implications on clinical and patient-reported outcomes for the foreseeable future. Here we have reviewed and integrated literature supporting how both the immunologic and psychologic vulnerability of TCT patients renders them particularly susceptible to adverse biobehavioral sequelae associated with Covid-19 infection, restrictions, and stressors.

Psychosocial factors associated with Covid-19 pandemic conditions activate intersecting psychobiological processes that lead to alterations in neural and endocrine pathways, predominately through SNS and HPA axis signaling. Dysregulation of these signaling pathways adversely affect cellular and immune function, increase inflammation, and are associated with worse cancer and TCT outcomes. Responses to both the stressor and infectivity of Covid-19 share overlapping pathophysiology modalities, particularly with inflammation. Rigorous translational investigation needs to focus on further delineating the mechanisms and clinical outcomes of the biobehavioral overlap to inform risk-stratification and targeted interventions. Patients’ emotional responses to stressors associated with Covid-19 should be directly addressed, and pharmacologic and behavioral interventions targeting end organ biologic effects secondary to biobehavioral processes should also continue to be investigated with the goal of mitigating adverse cancer outcomes.

As recently described in a publication from the American Society for Transplantation and Cellular Therapy Biobehavioral Research Special Interest Group (53), it is essential for the biobehavioral research community to unite on initiatives and priorities to move the science forward in a deliberate fashion for optimal outcomes. By integrating knowledge from prior biobehavioral work, we can test informed hypotheses regarding psychological and immunological responses to Covid-19 among TCT patients, thus promoting post-pandemic recovery and preparedness for future pandemics or other significant stressors.

## Author Contributions

All authors substantially contributed to the conception and design of the work; participated in drafting and revising the work; have provided approval for publication of the content; and agree to be accountable for all aspects of the work.

## Conflict of Interest

The authors declare that the research was conducted in the absence of any commercial or financial relationships that could be construed as a potential conflict of interest.

## Publisher’s Note

All claims expressed in this article are solely those of the authors and do not necessarily represent those of their affiliated organizations, or those of the publisher, the editors and the reviewers. Any product that may be evaluated in this article, or claim that may be made by its manufacturer, is not guaranteed or endorsed by the publisher.
